# Benralizumab for adults with rare and off-label eosinophilic disorders: a 52-week prospective, single-center study

**DOI:** 10.3389/fimmu.2025.1702989

**Published:** 2025-10-23

**Authors:** Aviv Talmon, Oded Shamriz, Limor Rubin, Yaarit Ribak, Iris Aynor, Adam Nevo, Anna Elia, Meitav Ben Sion, Esther Forkosh, Alon Y. Hershko, Yuval Tal

**Affiliations:** ^1^ Allergy and Clinical Immunology Unit, Department of Medicine, Hadassah Medical Organization, Faculty of Medicine, Hebrew University of Jerusalem, Jerusalem, Israel; ^2^ The Lautenberg Center for Immunology and Cancer Research, Institute of Medical Research Israel-Canada, Faculty of Medicine, Hebrew University of Jerusalem, Jerusalem, Israel; ^3^ Department of Radiology, Hadassah Medical Center, Faculty of Medicine, Hebrew University of Jerusalem, Jerusalem, Israel; ^4^ Department of Pathology, Hadassah Medical Center, Faculty of Medicine, Hebrew University of Jerusalem, Jerusalem, Israel; ^5^ Department of Gastroenterology, Hadassah Medical Center Faculty of Medicine, Hebrew University of Jerusalem, Jerusalem, Israel

**Keywords:** benralizumab, IL-5, eosinophilic disorders, basket trial, eosinophil

## Abstract

**Background:**

Rare eosinophilic disorders are challenging to manage due to their heterogeneity and lack of targeted therapies. Benralizumab, an anti-IL-5 receptor monoclonal antibody approved for the treatment of severe eosinophilic asthma and eosinophilic granulomatosis with polyangiitis (EGPA), has not been systematically studied in other eosinophilic conditions.

**Objective:**

To assess the efficacy and safety of benralizumab in adults with rare, non-asthmatic eosinophilic disorders over 52 weeks.

**Methods:**

In this single-center, prospective, open-label study, 17 adults with diverse eosinophilic diseases received benralizumab 30 mg every 4 weeks for 24 weeks; responders continued up to 52 weeks. The primary endpoint was ≥50% reduction in peripheral eosinophil counts or tissue infiltration. Secondary outcomes included symptom improvement, reduced exacerbations, corticosteroid withdrawal, and safety.

**Results:**

Of the 19 enrolled patients, 17 initiated treatment. Sixteen achieved clinical resolution, and all showed complete peripheral eosinophil depletion. Corticosteroids were discontinued in all completers. One patient had a partial response, and one discontinued due to mild, unrelated liver enzyme elevation. No serious adverse events occurred. Relapses were observed after treatment cessation. Efficacy was demonstrated across heterogeneous conditions, including eosinophilic leukemia, folliculitis, vaginitis, and IgG4-related disease.

**Conclusion:**

Benralizumab is safe, well-tolerated, and effective in diverse rare eosinophilic disorders, enabling corticosteroid discontinuation and symptom control. These findings support its broader therapeutic potential and warrant further investigation.

## Highlights

- What is already known about this topic?

Benralizumab is approved for the treatment of severe eosinophilic asthma and eosinophilic granulomatosis with polyangiitis (EGPA). However, its efficacy in other rare eosinophilic disorders remains largely understudied.

- What does this article add to our knowledge?

This prospective study suggests that benralizumab is safe, well-tolerated, and effective across a spectrum of rare eosinophilic conditions, enabling corticosteroid withdrawal and symptom control. However, the study was single-center, open-label, and non-randomized and included a small, heterogeneous cohort, limiting generalizability and introducing potential biases.

- How this study impact current management guidelines?

Findings highlight benralizumab’s potential role beyond approved indications and provide rationale for larger multicenter trials to confirm its efficacy and safety in rare eosinophilic disorders.

## 1 Introduction

Eosinophilic inflammation is primarily driven by interleukin (IL)-5, a key cytokine involved in the pathogenesis of allergic and eosinophilic conditions (1). Its role has been extensively studied in both human and murine models (2, 3). Eosinophilia is a hallmark of several diseases, including asthma, eosinophilic fasciitis (EF), eosinophilic cellulitis (EC), and eosinophilic granulomatosis with polyangiitis (EGPA), where eosinophils induce tissue inflammation across various organ systems (1).

Benralizumab, a humanized monoclonal antibody targeting the IL-5 receptor α-subunit (IL-5Rα), represents a therapeutic approach for eosinophilic disorders. Inhibition of IL-5 signaling suppresses eosinophil maturation, chemotaxis, and tissue infiltration. Furthermore, benralizumab engages the FcγRIIIa receptor on natural killer (NK) cells, inducing swift eosinophil apoptosis via granzyme and perforin release, thereby directly depleting circulating and tissue eosinophils (4). Benralizumab has demonstrated significant efficacy in reducing eosinophilic inflammation and improving clinical outcomes in severe eosinophilic asthma and EGPA (5, 6).

While benralizumab is approved for severe eosinophilic asthma and EGPA, its potential benefits in rare eosinophilic disorders are still under investigation. Previous reports, including those from our group, highlight its efficacy in conditions such as drug reaction with eosinophilia and systemic symptoms (DRESS) syndrome (7), immune checkpoint inhibitor-induced eosinophilic adverse events (8), and a small cohort of rare eosinophilic disorders, including eosinophilic cystitis, EF, and chronic eosinophilic pancreatitis and cholangitis (9). A phase 2 clinical trial for platelet-derived growth factor receptor alpha (PDGFRA)-negative hypereosinophilic syndrome (HES) has further demonstrated sustained clinical and laboratory responses, suggesting that benralizumab may offer therapeutic advantages in a broader range of eosinophilic conditions (10).

Given the rarity of these disorders and the small patient populations within each category, a “basket trial” methodology, as previously proposed by our group (9), may be an effective approach to assess benralizumab’s utility across diverse eosinophilic conditions. In this study, we aim to evaluate the laboratory and clinical efficacy of benralizumab in patients with non-asthma, rare eosinophilic disorders, addressing a critical gap in therapeutic options for these patients.

## 2 Methods

### 2.1 Study population and design

This prospective, open-label study (IRB number: HMO-0758-20) enrolled adult patients (aged 18 years and older) treated at the Allergy and Clinical Immunology Unit of Hadassah Medical Center, Jerusalem, Israel, between 2021 and 2023. Both outpatients and hospitalized individuals meeting the inclusion criteria based on clinical symptoms and evidence of eosinophilic-derived end-organ damage were included. Participants received benralizumab 30 mg every 4 weeks, with treatment lasting 24 weeks, during which six subcutaneous doses were administered. Patient evaluations were conducted by two senior physicians. If treatment was deemed unsuccessful, benralizumab was discontinued. Responders, defined as patients demonstrating clinical improvement, continued therapy for a total of 52 weeks. Non-responders were identified as those with persistent clinical symptoms and laboratory markers of active inflammatory disease within the initial 24 weeks.

### 2.2 Study objectives

A reduction of ≥50% in peripheral absolute eosinophil count (AEC) or tissue infiltration was defined as the primary endpoint, in line with previously published trials. For example, in a study of benralizumab in PDGFRA-negative HES, the primary endpoint was a ≥50% reduction in AEC at week 12 (10). Secondary goals included assessing symptom improvement through a reduction in the frequency of disease exacerbations and hospitalizations compared to baseline and a decrease in systemic corticosteroid use after initiating benralizumab treatment. Baseline corticosteroid dose and treatment duration were determined by a senior clinical immunologist at study onset. Following the start of benralizumab therapy, corticosteroid doses were tapered. Individualized clinical and laboratory markers were monitored to evaluate patient response to corticosteroid reduction and detect potential flare-ups following treatment initiation.

Disease-specific secondary endpoints were established for each eosinophilic disorder (detailed in **Supplementary Table 1**). Safety assessments included monitoring vital signs, clinical chemistry and hematology parameters, physical examinations, and documentation of adverse events reported by patients or investigators.

### 2.3 Inclusion criteria

Eligible patients demonstrated both laboratory and clinical evidence of eosinophil-driven disorders. Laboratory evidence included peripheral AEC ≥1,500 cells/µL within 5 years of diagnosis or a tissue biopsy obtained within 5 years showing eosinophil-related disease, irrespective of peripheral eosinophil count. For previously treated patients, a baseline tissue biopsy was obtained near study initiation. For untreated patients, biopsies taken at diagnosis served as the baseline. Histopathological diagnoses required increased eosinophil levels in tissue specimens, as determined by pathologists using accepted diagnostic criteria, with thresholds varying by disease and tissue type. A negative stool PCR test for parasites was mandatory for enrollment in the study. Clinical evidence included confirmed diagnoses of eosinophil-related diseases such as EF, eosinophilic colitis, eosinophilic pneumonia, eosinophilic bronchitis, or other conditions where eosinophils were identified as key contributors to pathogenesis, as determined by two senior clinical immunology specialists.

Patients were categorized as steroid-naive, steroid-resistant, or steroid-dependent. Steroid-naive patients had received corticosteroid treatment for ≤14 days post-diagnosis. Steroid-resistant patients had persistent or worsening end-organ damage and active inflammatory markers despite >14 days of corticosteroid treatment. Steroid-dependent patients achieved reasonable disease control but could not taper corticosteroid doses over 24 weeks and exhibited corticosteroid-related side effects such as diabetes, hypertension, or bone disease.

### 2.4 Exclusion criteria

Exclusion criteria included pregnancy, confirmed by a positive urinary or serum β-human chorionic gonadotropin test, or breastfeeding. Patients with eosinophilic esophagitis (EoE), non-EGPA, or HES who were eligible for benralizumab phase III trials were also excluded. Additional exclusions encompassed secondary eosinophilia due to parasitic infection or malignancy, known hypersensitivity to benralizumab or any of its components, positive human immunodeficiency virus (HIV) status, and drug or alcohol abuse within the year preceding enrollment. Patients with creatinine clearance below 30 mL/min (per the Cockcroft–Gault formula) or those dependent on dialysis were ineligible, as were any individuals whom the investigators judged unable to comply with study requirements.

### 2.5 Ethical review of the study

The study received approval from the institutional review board of Hadassah Medical Organization (IRB number: HMO-0758-20). All participants provided written informed consent prior to enrollment in accordance with the IRB guidelines.

## 3 Results

### 3.1 Baseline characteristics of the patients

**Figure 1:**
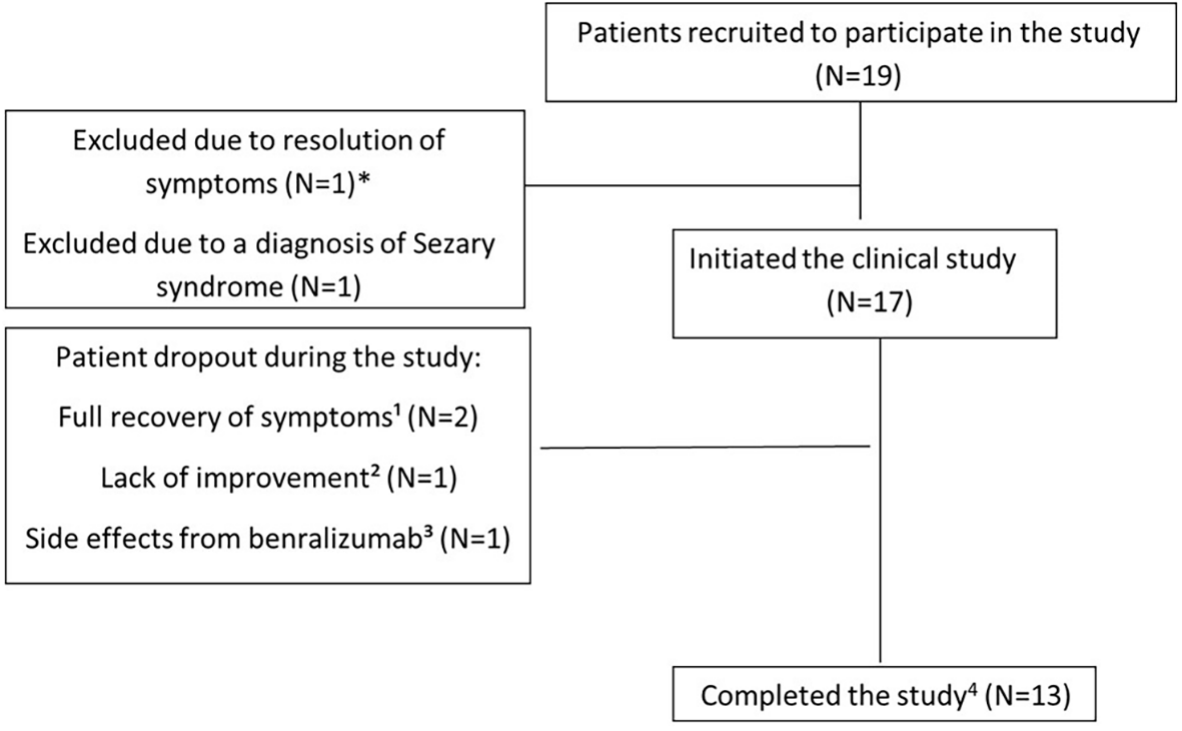
Schematic model of the study. *One patient with eosinophilic pneumonia withdrew steroids without relapse before study initiation and was excluded. ¹One patient with familial Mediterranean fever and eosinophilic abscess (mediastinal mass, skin nodules) did not improve after four benralizumab injections. ²One patient with eosinophilic dermatitis developed gradual liver enzyme elevation after five doses. ³One patient with sulfasalazine-induced acute eosinophilic pneumonia achieved complete resolution after two doses; another with eosinophilic cellulitis achieved complete resolution after seven doses. ^4^The protocol allowed investigators to administer an additional benralizumab injection; thus, patients received 14 or 15 doses.

**Figure 2:**
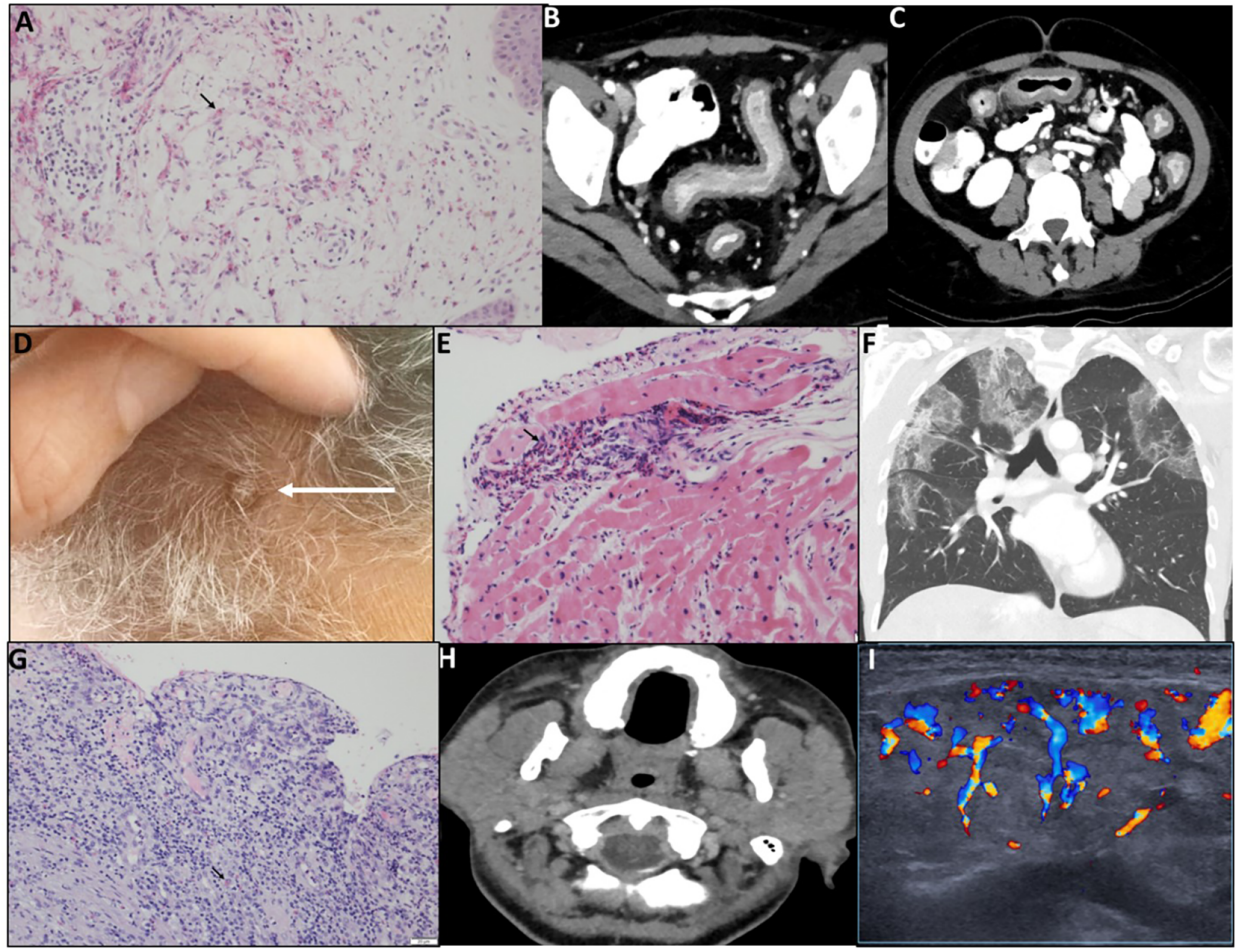
Representative patients demonstrating different manifestations of rare eosinophilic disorders. **(A)** P1, Wells syndrome. Skin biopsy (H&E) shows full-thickness perivascular, periadnexal, and interstitial eosinophilic infiltrates with dermal degranulation (arrow). **(B, C)** P6, eosinophilic colitis. Pre-treatment CT shows diffuse colonic wall thickening, mucosal enhancement, and mild surrounding fat stranding; no abscess or fistula. **(D)** P8, neutrophilic dermatitis with eosinophilic sinusitis. Scalp lesions exhibit erythema and scaling. **(E)** P17, EGPA with eosinophilic carditis. Myocardial biopsy shows interstitial and endocardial eosinophilic infiltrates, focal cardiomyocyte damage, and a small non-caseating granuloma; vessels intact (arrow). **(F)** P10, chronic eosinophilic pneumonia. Chest CT shows bilateral peripheral ground-glass opacities, predominantly upper lobes; coronal view demonstrates “reverse bat wing” pattern. **(G)** P16, eosinophilic vaginitis. Vaginal ulcer biopsy shows chronic neutrophil-rich infiltrate with eosinophils (arrow); epithelium detached at ulcer margins. **(H, I)** P11, eosinophilic bronchitis and sialoadenitis. Neck CT shows enlarged, edematous parotid and submandibular glands with fat stranding **(H)**; parotid US demonstrates edema and hypervascularity **(I)**.

**Figure 3:**
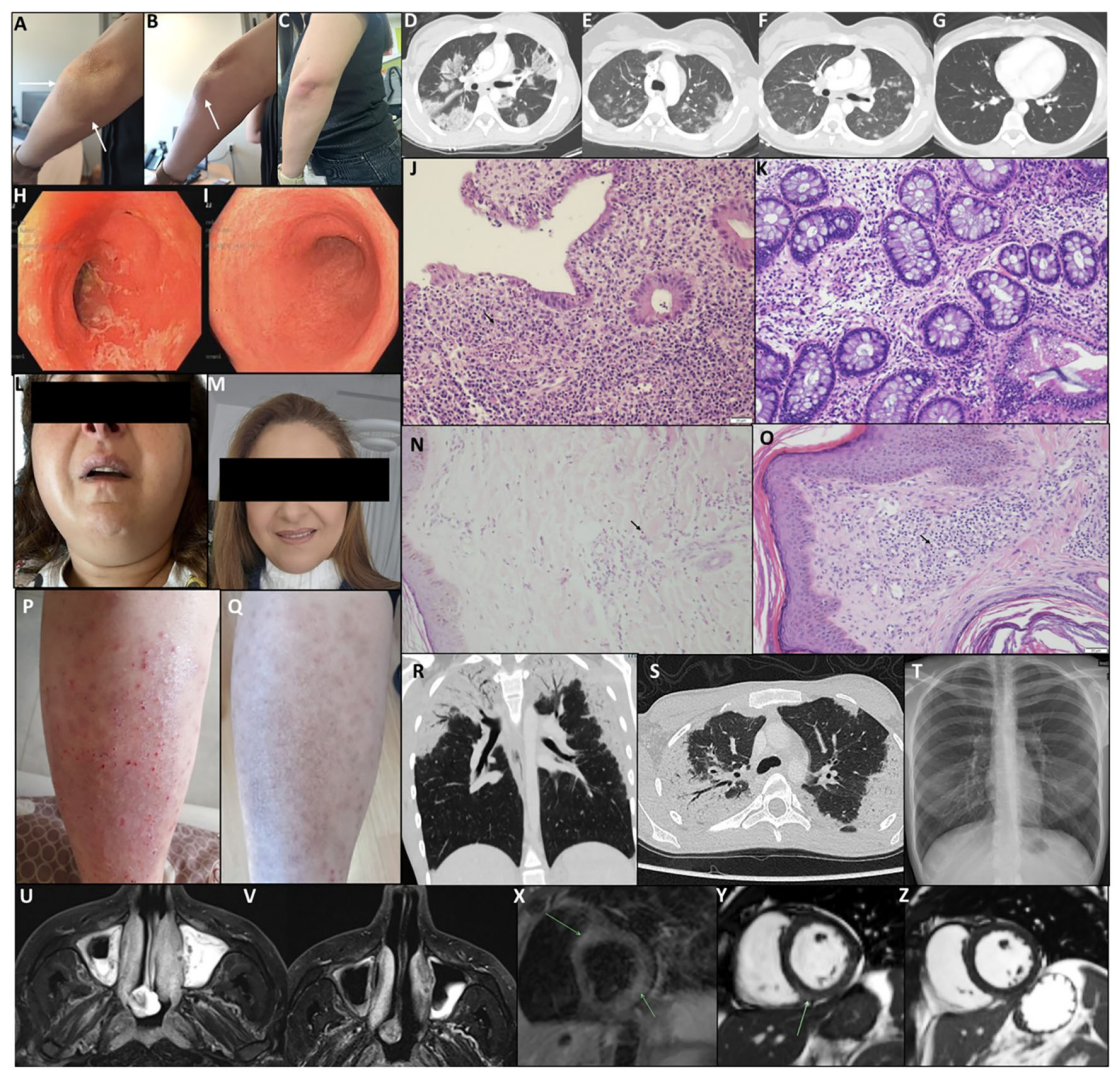
Clinical, histological, and radiological follow-up during treatment with benralizumab. **(A–C)** P1, eosinophilic cellulitis (Wells syndrome). Pre-treatment lesion inferior to the left elbow **(A, B)** shows erythema and inflammation (arrows); post-treatment **(C)** demonstrates marked improvement. **(D–G)** P3, chronic eosinophilic pneumonia. Chest CT pre-treatment **(D)** shows bilateral peripheral ground-glass opacities; after the first dose **(E, F)**, marked improvement; after three doses **(G)**, complete resolution. **(H–K)** P6, eosinophilic colitis. Sigmoidoscopy pre-treatment **(H)** shows friable, erosive mucosa; post five doses **(I)**, normal mucosa. Rectal biopsy pre-treatment **(J)** shows eosinophilic infiltration, cryptitis, and glandular distortion (arrow); post-treatment **(K)** shows mild residual inflammation. **(L, M)** P11, eosinophilic bronchitis and sialoadenitis. Pre-treatment bilateral parotitis **(L)**; post single dose **(M)** shows normal appearance. **(N, O)** P8, neutrophilic dermatitis and eosinophilic sinusitis. Pre-study biopsy **(N)** shows dermal neutrophilic/eosinophilic infiltrates (arrow); on-study biopsy **(O)** shows mild perivascular mononuclear infiltrates with occasional eosinophils; lesions resolved under treatment. **(P, Q)** P13, non-specific eosinophilic dermatitis. Pre-treatment eczematous plaques **(P)**; post two doses **(Q)**, rash resolved with residual hyperpigmentation. **(R–T)** P14, salazopyrin-induced acute eosinophilic pneumonia. Chest CT at diagnosis **(R, S)** shows bilateral peripheral GGO; post three doses **(T)**, chest X-ray normal. **(U, V)** P15, eosinophilic sinusitis with nasal polyposis. MRI T2 pre-study **(U)** shows severe sinus disease; post seven doses **(V)** shows improvement. **(X–Z)** P17, severe EGPA with eosinophilic carditis. Cardiac MRI pre-treatment **(X)** shows myocardial edema; short-axis SSFP **(Y)** shows mid-wall LGE consistent with myocarditis; post-treatment **(Z)** shows resolution of LGE.

The clinical characteristics of the patients are summarized in **Table 1**. A total of 19 patients with various eosinophilic disorders were enrolled; however, two were excluded before initiating benralizumab. One was excluded due to symptom resolution. The other was initially enrolled with suspected eosinophilic cellulitis, but subsequent biopsy review confirmed Sézary syndrome. As Sézary syndrome is not a primary eosinophilic disorder and evaluation of benralizumab in this context was beyond the scope of the trial, the patient was excluded (**Figure 1**). All patients previously treated with biologics or targeted agents underwent adequate washout before enrollment. Patients 1 and 8 had received mepolizumab, which has a half-life of approximately 20 days, but discontinued treatment 10 and 24 months prior, respectively, well beyond the recommended five half-lives. Patient 6 had received vedolizumab 15 months before enrollment, and patient 7 discontinued imatinib 3 months prior, exceeding the clearance period of its ~40-h half-life. Thus, all prior therapies were stopped sufficiently in advance to prevent residual activity or confounding effects. Among the 17 patients who proceeded with the study and received benralizumab, 10 were women, 3 were Arab, and 14 were Jewish. The mean age at diagnosis was 45.26 years (range: 1.5–100), and the mean age at enrollment was 49.06 years (range: 18–100). The diagnoses of eosinophilic disorders were based on clinical manifestations combined with evidence of eosinophilia in either peripheral blood or affected tissues. The clinical features of each case are detailed in **Table 1**. Among the 17 patients, five (29.4%) had eosinophilic disorders involving the skin, including eosinophilic cellulitis (*n* = 1) (11), non-specific eosinophilic dermatitis (*n* = 2), eosinophilic folliculitis (Ofuji disease) (*n* = 1), and Kimura’s disease (*n* = 1). Four patients (23.5%) exhibited respiratory system involvement, with diagnoses of chronic eosinophilic pneumonia (CEP; *n* = 2), eosinophilic bronchitis (*n* = 1), and salazopyrin-induced acute eosinophilic pneumonia (AEP; *n* = 1). Two patients (11.8%) had sinus involvement, including eosinophilic sinusitis, one of whom was subsequently diagnosed with IgG4-related disease (IgG4-RD). Another two patients (11.8%) had eosinophilic disorders affecting the genitourinary tract—eosinophilic cystitis (*n* = 1) and eosinophilic vaginitis (*n* = 1). Additionally, there was one patient (5.9%) each with soft tissue involvement (eosinophilic mediastinal mass, *n* = 1), gastrointestinal involvement (eosinophilic colitis, *n* = 1), and cardiovascular involvement (severe EGPA with eosinophilic carditis, *n* = 1). Finally, one patient (5.9%) presented with a primary myeloid eosinophilic disorder. FISH analysis demonstrated a non-classical PDGFRB-5q32 translocation in 9% of cells, whereas 4% of cells exhibited loss of one PDGFRB copy. Clinical and histological images of the enrolled subjects are depicted in **Figures 2** and **3**, respectively.

**Table 1:** Clinical characteristics of the study cohort.

Patient	Age at diagnosis (years)	Age at trial enrollment (years)	Sex	Diagnosis	Clinical manifestations	Medical history	Maximum pre-trial absolute eosinophil count (0-0.5 10^9^/L)	Eosinophils in other sites	Immuno-modulatory treatment*	Systemic GCs naïve/resistant /dependent
1^11^	0.5	32	F	Eosinophilic Cellulitis (Wells syndrome)	Multiple skin lesions	None	1.1	+/ Skin	Mepolizumab** Dapsone Cyclosporine Colchicine	Dependent
2	44	48	M	Mediastinal mass with eosinophilic abscess	Chest pain and dyspnea	FMF	0.3	+/ Abscess in the SCJ	Colchicine Anakinra MTX Azathioprine	Dependent
3	17	21	F	CEP	Dyspnea, cough	None	4.1	–	None	Naïve***
4	100	100	F	Eosinophilic Cystitis	Severe dysuria	OP, Hypothyroidism CRF, Diverticulitis, Chondrodermatitis	2.9	+ / Urine	None	Resistant
5	83	83	M	Non- specific eosinophilic dermatitis	Itchy rash	SBO , PE Anal fissure Thromboflebitis Malignant neoplasm appendix vermiformis	0.8	+/ Skin	None	Dependent
6	26	28	F	Eosinophilic Colitis	Abdominal Pain, Diarrhea, fever. Tenesmus	UC CMV colitis	2.8	+/ Rectum	Infliximab Vedolizumab^+^	Dependent
7	83	84	F	Eosinophilic leukemia	Pruritus, Elevated live enzyme, weight loss	Dyslipidemia, HTN, OP, AMD,	4.5	+ / BM	Imatinib^++^	Dependent
8	62	64	M	Non- specific neutrophilic dermatitis, Eosinophilic sinusitis	Chronic urticaria, angioedema	Dyslipidemia, CRF	10	+/ BM,	Mepolizumab	Dependent
9	60	60	M	Kimura's Disease	Submandibular lymphadenopathy, Skin nodules, rash	Diabetes, dyslipidemia, CVD	1.3	+/ Lymph node	None	Naive
10	35	42	F	CEP	Dyspnea	Severe GCs-induced OP; Multiple pathological fractures	3.5	+/ BAL^#^	None	Dependent
11	44	44	F	Eosinophilic sialoadenitis; Eosinophilic bronchitis	Acute parotitis	Hypothyroidism, atopic dermatitis, allergic rhinitis	2.5	–	None	Dependent
12	25	30	M	Eosinophilic folliculitis (Ofuji disease)	Pruritus	PV, smoking, exertional asthma	2.0	+ / BM, skin	None	Dependent
13	51	52	F	Non- specific eosinophilic dermatitis	Pruritus	Asthma	0.22	+/ Skin	Topical Tacrolimus and GCs, Phototherapy	Naive
14	18	18	F	Salazyopyrin-induced AEP	Dyspnea	UC, Hypothyroidism	1.2	+/ BAL	None	Naive
15	59	62	M	Eosinophilic sinusitis and nasal polyposis (IgG4-related disorder)	nasal polyposis, dermatitis, sinusitis, orbital mass, asthma	Hepatitis B virus carrier Eosinophilic Hepatitis	20.2	+/ Liver, BM, sinuses	MTX	Dependent
16	26	28	F	Eosinophilic vaginitis	Dyspareunia	Recurrent pneumonia ^#,+^, Asthma	0.29	+/ Vagina	None	Naive
17	35	38	M	EGPA with eosinophilic carditis	Sinusitis, carditis, Peripheral neuropathy	Asthma	17.6	+/ Heart	None	Naive

F, female; M, male; J, Jew; A, Arab; GCs, glucocorticosteroids; CEP, chronic eosinophilic pneumonia; OP, osteoporosis; HTN, hypertension; CRF, chronic renal failure; SCJ, sternoclavicular joint; BM, bone marrow; CVD, coronary vascular disease; CEP, chronic eosinophilic pneumonia; BAL, bronchoalveolar lavage; SBO, small bowel obstruction; AMD, age-related macular degeneration; PV, polycythemia vera; AEP, acute eosinophilic pneumonia; MTX, methotrexate; FMF, familial Mediterranean fever; CHF, congestive heart failure; UC, ulcerative colitis; CMV, cytomegalovirus; PBC, primary biliary cirrhosis; EGPA, eosinophilic granulomatosis with polyangiitis.

*Washout periods for patients 1, 6, 7, and 8 were 10, 15, 3, and 24 months, respectively, each exceeding the corresponding biologic agent’s half-life. **Given at 100 mg every 4 weeks at age 27, discontinued after two injections due to headaches and fatigue. ***Benralizumab was initiated together with GCs, enabling tapering of GCs.

+Given for an initial diagnosis of UC.

++Given for a non-classical PDGFRB-5q32 translocation detected in 9% of cells.

#Bronchoalveolar lavage (BAL) showed 83% eosinophils.

#,+No BAL available; however, the patient presented with patchy infiltrates responsive to GCs, suggesting recurrent eosinophilic pneumonia.

The mean (range) AEC in peripheral blood prior to treatment initiation was 4.43 (0.22–20.2) × 10^9^/L, compared to the normal range of 0–0.5 × 10^9^/L. Tissue eosinophilic infiltration was noted in 15 (88.23%) patients. In terms of corticosteroid use, 10 patients (58.8%) were classified as steroid-dependent, 6 (35.3%) were steroid-naive, and 1 (5.9%) was steroid-resistant. Five (29.4%) patients were treated with other biologicals prior to study enrollment. Representative clinical, histological, and radiological characteristics of the patients before study enrolment are presented in **Figure 2**.

### 3.2 Safety of benralizumab treatment in the study cohort

During the 52-week follow-up period, 4 of the 17 patients who started benralizumab treatment have discontinued the study (**Figure 1**). Two patients withdrew from the study due to complete symptom resolution (P13 and P14), while one patient (P2) discontinued treatment because of insufficient improvement. Another patient (P5) was found to have mildly elevated hepatocellular enzyme levels during follow-up [aspartate transaminase (AST): 74 IU/L (normal range: 10–40 IU/L), alanine transaminase (ALT): 72 IU/L (normal range: 7–56 IU/L), alkaline phosphatase (ALKP): 290 IU/L (normal range: 44–147 IU/L), and gamma-glutamyl transferase (GGT): 147 IU/L (normal range: 9–48 IU/L)]. Although a causal relationship with benralizumab could not be confirmed, the treatment was discontinued. Liver enzyme levels gradually normalized over the course of approximately 1 year. However, a subsequent gradual increase was observed, peaking 2 years later at levels higher than those recorded during the study. These findings suggest that drug-induced liver injury (DILI) secondary to benralizumab did not occur in our trial. According to the Common Terminology Criteria for Adverse Events (CTCAE), this event corresponded to grade 1. No other treatment-related or unrelated adverse events, of any grade, were observed.

At the last follow-up visit, impaired renal function was noted in two patients (P4 and P8); however, elevated creatinine levels had already been documented prior to treatment initiation, indicating pre-existing chronic renal failure unrelated to benralizumab.

No serious adverse events (SAEs), suspected unexpected serious adverse reactions (SUSARs), or benralizumab-related mortality were reported. Complete blood counts, liver enzyme levels, and creatinine values from the final follow-up visit are provided in **Supplementary Table 2**.

### 3.3 Efficacy of benralizumab in the study cohort

#### 3.3.1 Clinical and laboratory outcomes

Outcomes of the treated patients are summarized in **Table 2**. Representative patients with clinical resolution are presented in **Figure 3**. Comparison between maximal pre-treatment AEC and AEC at the last follow-up visit demonstrated a significant reduction with benralizumab treatment, from a mean of 4.43 × 10^9^/L (0.22–20.2) to 0.018 × 10^9^/L (0–0.1) (paired *t*-test, *p* = 0.0122; 95% confidence interval: 0.9021–6.2766). Disease exacerbations during the study period were observed in only two patients, both associated with missed visits. No response to treatment was noted in one patient (P2) with a mediastinal mass and an eosinophilic abscess. Partial clinical response was noted in two patients (P9 and P12 with Kimura’s and Ofuji diseases, respectively). All patients who completed the trial have successfully discontinued corticosteroid treatment, including P16, who had eosinophilic vaginitis and ceased chronic intravaginal corticosteroid injections used to manage her vaginal lesions. Patient P11, who presented with eosinophilic sialoadenitis and bronchitis, had normal spirometry results but an elevated fractional exhaled nitric oxide (FeNO) level of 58.5 ppb at diagnosis. At her most recent follow-up, her FeNO had decreased to 30 ppb. One patient (P14) received only two benralizumab injections before discontinuing treatment due to the complete resolution of drug-induced AEP. Five patients (P4, P6, P7, P11, and P14) experienced no exacerbations from the study’s conclusion until the manuscript’s preparation. Furthermore, these patients were not administered the investigational drug during this timeframe. P4’s dysuria resolved, and the patient died 1 year post-study from aspiration pneumonia at the age of 101. All remaining patients experienced exacerbations after the trial’s conclusion and the discontinuation of benralizumab. Following observed clinical deterioration, patients P1, P9, P10, P13, and P16 were administered the investigational drug under AstraZeneca’s pharmaceutical access program.

**Table 2:** Treatment, course, and clinical outcome of patients treated with benralizumab in the study cohort.

Patient	Diagnosis	Diagnosis	Clinical outcome on last follow-up			Laboratory outcome on last follow-up	
		Number of disease exacerbations during the study period	Number of hospitalizations during the study period.	Systemic GCs	Disease-specific clinical efficacy endpoints	Peripheral blood eosinophil count on last follow-up visit* (cells x10^3^)	Evidence of resolution of eosinophilic infiltration in tissue Biopsies**
1	Eosinophilic Cellulitis (Wells syndrome)	0	0	No GCs	CR of skin lesions	0	NA
2	Mediastinal mass with eosinophilic abscess	0	0	No GCs	No response	0	NA
3	CEP	1***	1***	No GCs	CR of cough and dyspnea	0.1	NA
4	Eosinophilic Cystitis	0	0	No GCs	CR of urinary symptoms	0	NA
5	Non- specific eosinophilic dermatitis+	0	0	No GCs	No response	NA	NA
6	Eosinophilic Colitis	0	0	No GCs	CR of GI symptoms	0	Repeated colonoscopy: chronic focally mildly active inflammatory bowel disease
7	Eosinophilic leukemia	0	0	No GCs	CR of pruritus and regained 5 kg after 15 kg weight loss	0	NA
8	Non- specific neutrophilic dermatitis, Eosinophilic sinusitis	1	0	No GCs	CR of skin lesions	0.01	NA
9	Kimura's Disease++	0	0	No GCs	Mild pruritus, CR of rash, decrease in size of SC nodules	0	NA
10	CEP	0	0	No GCs	Improvement of cough , CR of dyspnea	0.03	NA
11	Eosinophilic sialoadenitis; Eosinophilic bronchitis	0	0	No GCs	CR of bronchitis and sialoadenitis	0.06	NA
12	Eosinophilic folliculitis (Ofuji disease)	0	0	No GCs	Improvement in pruritus – grade 2/10	0	NA
13	Non- specific eosinophilic dermatitis	0	0	No GCs	CR	0	NA
14	Salazyopyrin-induced AEP*+	0	0	No GCs	CR	0	NA
15	Eosinophilic sinusitis and nasal polyposis IgG4-related disorder))	0	0	No GCs	CR	0	NA
16	Eosinophilic vaginitis	0	0	Discontinued topical GCs vaginal injections	CR	0	NA
17	EGPA with eosinophilic carditis	0	0	No GCs	Stable	0.08	NA

GI, gastrointestinal; GCs, glucocorticosteroids; CEP, chronic eosinophilic pneumonia; AEP, acute eosinophilic pneumonia; EGPA, eosinophilic granulomatosis with polyangiitis; CR, complete resolution; WNL, within normal limits; NA, data not available.

*≥50% decrease from baseline count after 24 weeks of benralizumab treatment.

**≥50% decrease from baseline biopsies within 24 weeks of benralizumab treatment.

***A single exacerbation occurred 6 months into the study during treatment discontinuation; therapy was resumed after 3 months.

+Benralizumab was discontinued due to elevated liver enzymes.

++Exacerbation occurred before the last dose due to a missed injection.

*+Received only two benralizumab injections; treatment was discontinued following complete clinical resolution.

#### 3.3.2 Histological and radiological evidence for benralizumab efficacy

**Figure 4:**
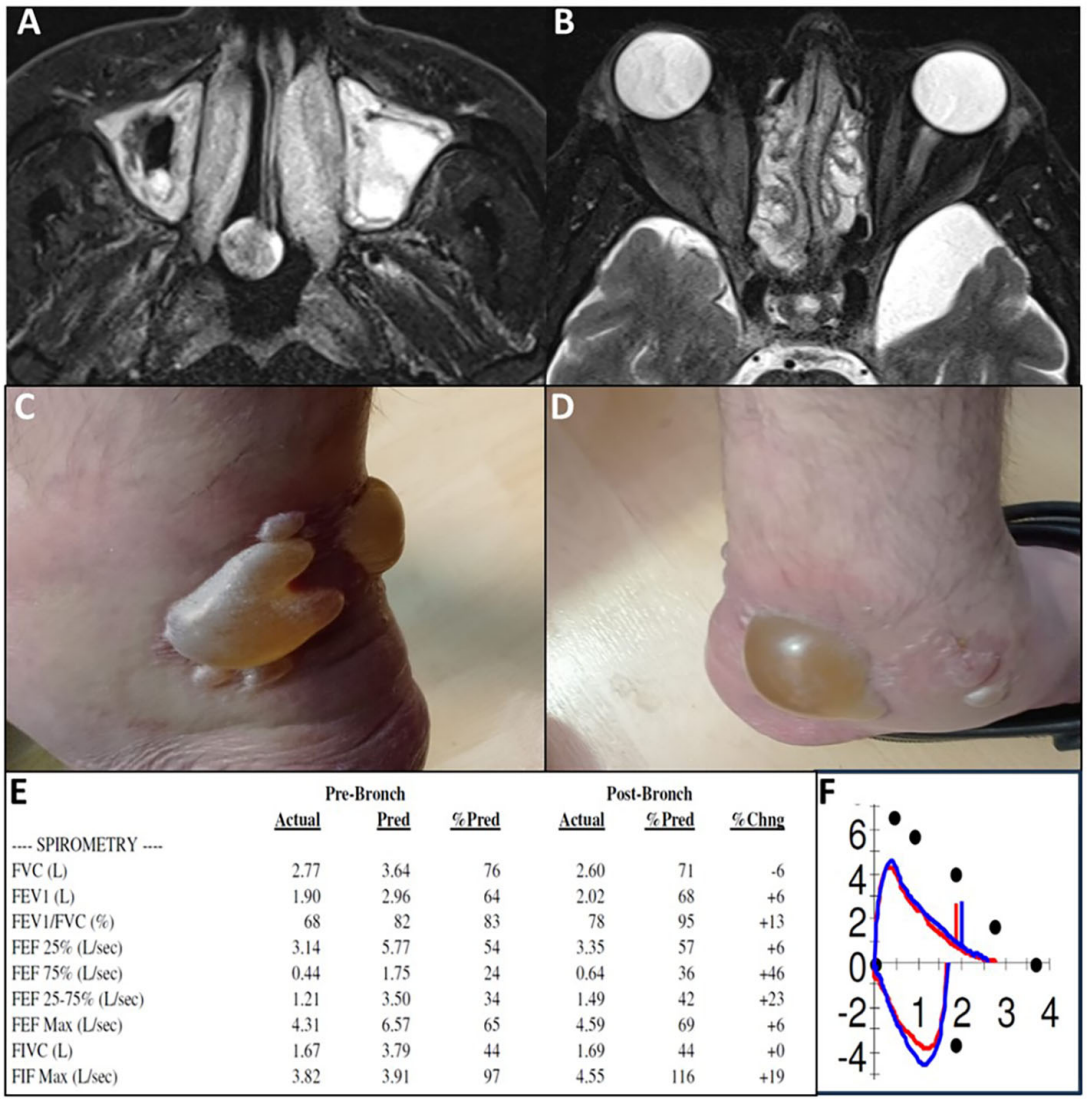
Recurrence of diseases following study completion and benralizumab discontinuation. **(A, B)** P15, eosinophilic sinusitis with nasal polyposis. MRI 2 months post-study shows recurrence of severe sinus disease (T2 axial images). **(C, D)** P17, severe EGPA with eosinophilic carditis. Bilateral ankle bullae observed 6 months post-study during EGPA exacerbation with worsening asthma and elevated inflammatory markers; fluid analysis shows 600 eosinophils. **(E, F)** P10, steroid-dependent chronic eosinophilic pneumonia. Pulmonary function tests 5 months post-study demonstrate combined obstructive and restrictive lung disease (FEV1/FVC 68%, FEV1 64% predicted, FVC 76% predicted). Flow-volume loop **(F)** shows a concave expiratory limb indicating obstruction and reduced loop size indicating restriction, consistent with PFT results.

Repeated tissue histology under treatment was available for one patient. Patient 6 initially presented with eosinophilic colitis, which showed significant improvement on follow-up biopsies, leaving only mild chronic active colonic inflammation. Further evidence of treatment efficacy was observed through repeated sigmoidoscopy, chest computed tomography (CT), magnetic resonance imaging (MRI), and cardiac MRI scans (**Figure 3**). However, disease exacerbations were noted after the discontinuation of benralizumab at the end of the trial. **Figure 4** illustrates representative cases of patients’ eosinophilic disorder exacerbations following the conclusion of the study.

## 4 Discussion

In this 52-week prospective “basket” trial, we evaluated the efficacy and safety of benralizumab in patients with rare eosinophilic disorders. To date, no double-blind randomized controlled trials are planned for these rare eosinophilic disorders, and our study contributes a unique perspective by providing prospective data with 52 weeks of follow-up. However, these findings represent exploratory, hypothesis-generating observations and should be interpreted with caution until validated in larger, controlled trials.

Benralizumab showed a favorable safety profile in our cohort, with only one patient discontinuing treatment due to mildly elevated liver enzymes later deemed unrelated to the intervention. No treatment-related adverse events or deaths occurred. These findings align with clinical trials and real-world studies, which consistently report a high safety profile. Long-term data from a 5-year follow-up in asthma patients demonstrated low rates of adverse and serious adverse events without new safety reports (12). This is further supported by a meta-analysis of eight RCTs of benralizumab in severe eosinophilic asthma (13).

In terms of efficacy, 16 of 17 patients achieved clinical resolution, with reductions in both eosinophil-induced organ damage and systemic manifestations. Steroid-dependent patients were able to discontinue corticosteroids without an increase in exacerbations or hospitalizations. Importantly, AEC significantly decreased in all patients. In one patient, repeat biopsy demonstrated a marked reduction in tissue eosinophil infiltration, mirroring the decline in circulating eosinophils. Additionally, serial CT and MRI imaging confirmed the clinical improvements observed during treatment with benralizumab.

Our study contributes preliminary data to several existing paradigms related to eosinophilic disorders. One major paradigm concerns the challenges of evaluating eosinophil effector involvement to accurately characterize their mechanistic role. Eosinophilic disorders can mimic other conditions, leading to potential misdiagnoses. For instance, one patient (P6) in our study was initially diagnosed with ulcerative colitis (UC) but was unresponsive to conventional therapies. Despite pathology consistent with UC, significant peripheral eosinophilia prompted re-evaluation, ultimately revealing eosinophilic colitis. The patient’s positive response to benralizumab highlights the importance of considering eosinophilic disorders in cases of treatment-resistant inflammatory bowel disease (IBD). Peripheral eosinophilia is common in IBD, occurring in 13% of patients, with a higher prevalence in UC than in Crohn’s disease, and has been identified as a predictor of severe disease (14). Diagnosing eosinophilic colitis remains challenging, as eosinophilic infiltration may extend beyond the mucosa, making detection difficult with standard biopsy techniques (15). Deep, invasive biopsies are often avoided in clinical practice. Non-invasive biomarkers, such as eosinophil-derived neurotoxin and Charcot–Leyden crystals detected in stool samples, may offer alternative diagnostic options (16).

Another paradigm addressed in our study was the efficacy of benralizumab both as a steroid-sparing treatment in chronic eosinophilic diseases and as a monotherapy in acute eosinophilic disorders. The utility of benralizumab as a steroid-sparing agent is exemplified by three distinct cases of eosinophilic pneumonia. In two patients with CEP, benralizumab enabled successful tapering and discontinuation of long-term corticosteroids. One patient (P10), who had been on steroids for 7 years and developed severe osteoporosis with spontaneous fractures, was able to discontinue steroids completely. In the second patient (P3), combining benralizumab with corticosteroids led to rapid induction of remission and near-immediate steroid discontinuation.

Notably, P14, who presented with salazopyrin-induced AEP, achieved full clinical recovery with benralizumab monotherapy, without any corticosteroid use. This unprecedented steroid-free outcome may reflect the rapid and profound eosinophil depletion induced by benralizumab. While this finding raises the possibility that, in acute settings where eosinophils are the primary drivers of pathology, benralizumab monotherapy could be sufficient, such an approach should be interpreted with caution. The observation may not extend to chronic eosinophilic diseases, where multiple immune pathways are involved, and requires confirmation in larger, controlled studies. Nevertheless, benralizumab could hold value for patients with contraindications to corticosteroids, especially in drug-induced cases where the offending agent has been discontinued.

Our study also identified a novel therapeutic application of benralizumab in IgG4-RD, as demonstrated by P15. This patient highlights a previously underappreciated role of eosinophils in IgG4-RD, challenging current treatment paradigms. Patient P15 initially presented with severe pansinusitis, nasal polyposis, an orbital mass, and asthma—all of which resolved with benralizumab. However, after therapy discontinuation, the patient experienced a significant disease flare requiring surgical interventions. Histopathological examination of the polyp tissue revealed marked eosinophilic infiltration (up to 150 eosinophils per high-power field), over 100 IgG4-positive plasma cells, and an elevated IgG4/IgG1 ratio exceeding 45%, alongside elevated serum IgG4 levels (806 mg/dL), confirming the IgG4-RD diagnosis (17). Notably, rituximab treatment worsened the patient’s condition, while eosinophil-targeted therapy was effective.

Eosinophilia is observed in 20%–40% of IgG4-RD patients and is associated with higher serum IgG4 levels, greater disease severity, and poorer treatment response. Pathophysiologically, elevated Th2 cytokine production stimulates B-cell-derived eotaxin-1 secretion, thereby promoting eosinophil recruitment and activation (18, 19). Eosinophils further contribute to fibrosis by secreting IL-13 and TGF-β, activating M2 monocytes, and supporting plasmablast survival through a proliferation-inducing ligand (APRIL), IL-6, IL-4, and IL-10 production. This patient highlights the complex interplay between lymphocytic and eosinophilic components in IgG4-RD and suggests that eosinophils may be key mediators of tissue damage.

Finally, our study provides early exploratory data, and while it suggests a potential role for benralizumab in eosinophilic leukemia and clonal eosinophilic diseases, these findings should be interpreted with caution and require confirmation in larger, controlled studies. Eosinophilic leukemia is a rare entity classified under “myeloid/lymphoid neoplasms with eosinophilia and tyrosine kinase gene fusions” (MLN-TK) according to the WHO and ICC criteria (20). Current treatments mainly involve tyrosine kinase inhibitors and bone marrow transplantation in refractory cases (21). IL-5 receptor expression commits myeloid progenitors to eosinophilic differentiation (22). In our study, we treated a patient (P7) with myeloid eosinophilic malignancy using benralizumab.

We hypothesize that benralizumab, through antibody-dependent cellular cytotoxicity (ADCC) targeting IL-5R-expressing cells, may eradicate malignant eosinophil progenitors, independent of specific tyrosine kinase mutations. To our knowledge, this is the first documented case of benralizumab use in eosinophilic leukemia, offering a novel, targeted therapeutic option. Furthermore, four other patients (P4, P6, P11, and P14) achieved sustained remission without relapse, suggesting that benralizumab may eliminate eosinophilic progenitor clones and potentially offer a curative approach in some eosinophilic diseases. Our study has several methodological limitations that should be considered when interpreting the results. First, this was a single-center trial with a small sample size (17 patients), which limits statistical power and generalizability. A further limitation is that we did not evaluate additional markers of benralizumab efficacy, such as serum cytokines, eosinophilic cationic protein (ECP), or FeNO, nor did we assess patients’ quality of life through standardized questionnaires. Accordingly, the findings should be regarded as preliminary and hypothesis-generating rather than definitive for clinical practice. Second, the open-label, non-randomized, single-arm design without a control or placebo group introduces a high risk of bias. This “basket trial” design was selected due to the rarity and heterogeneity of eosinophilic disorders; while not methodologically optimal, comparable single-arm basket trials have been successfully applied in other rare diseases and have contributed to the development of novel therapies (23, 24). Third, recruitment from a tertiary referral immunology unit may have introduced selection bias, as enrolled patients likely represented more severe disease phenotypes. In addition, sampling bias should be considered since patients with EoE and trial-eligible HES were excluded due to enrollment in other designated trials. Fourth, observer bias is possible because response was determined by physicians’ clinical assessment. To mitigate this, we complemented clinical observations with objective laboratory and radiological follow-up data. Fifth, histological confirmation was limited, and repeat tissue sampling was performed only when clinically indicated, reducing consistency in outcome assessment. Finally, the lack of long-term follow-up beyond remission precludes conclusions regarding the durability of efficacy and safety. Together, these limitations underscore the need for larger, multicenter, randomized controlled trials to validate and expand upon our findings.

In conclusion, benralizumab appears to demonstrate promising safety and efficacy across a range of rare eosinophilic disorders, thus offering novel therapeutic indications. Additional prospective trials are warranted to validate our results and expand its approved indications for these patient populations.

## Data Availability

The raw data supporting the conclusions of this article will be made available upon request from the corresponding author.

## Glossary

ADCC: antibody-dependent cellular cytotoxicity
AEP: acute eosinophilic pneumonia
ALK: alkaline phosphatase
ALT: alanine transaminase
APRIL: a proliferation-inducing ligand
AST: aspartate transaminase
CEP: chronic eosinophilic pneumonia
CT: computed tomography
DILI: drug-induced liver injury
DRESS: drug reaction with eosinophilia and systemic symptoms
EC: eosinophilic cellulitis
EF: eosinophilic fasciitis
EGPA: eosinophilic granulomatosis with polyangiitis
EoE: eosinophilic esophagitis
FeNO: fractional exhaled nitric oxide
FEV1: forced expiratory volume
FVC: forced vital capacity
GGT: gamma-glutamyl transferase
GGO: ground-glass opacities
H&E: hematoxylin and eosin
HES: hypereosinophilic syndrome
HIV: human immunodeficiency virus
IBD: inflammatory bowel disease
IgG4-RD: immunoglobulin g4-related disease
IL-5: interleukin-5
IL-5Rα: interleukin-5 receptor alpha subunit
IRB: Institutional Review Board
LGE: late gadolinium enhancement
MLNTK: myloid/lymphoid neoplasms with eosinophilia and tyrosine kinase gene fusions
MRI: magnetic resonance imaging
NK: natural killer
PDGFRA/PDGFRB: platelet-derived growth factor receptor alpha/beta
SAEs: serious adverse events
SSFP: steady-state free precession
SUSARs: suspected unexpected serious adverse reactions
UC: ulcerative colitis
WHO: World Health Organization
