# Supplementary material for: Benralizumab for adults with rare and off-label eosinophilic disorders: a 52-week prospective, single-center study
Source: Front Immunol. 2025 Oct 23;16:1702989. doi: 10.3389/fimmu.2025.1702989 (PMC12590485; doi:10.3389/fimmu.2025.1702989)
Supplement: Supplementary file 1 [file DataSheet1.docx]

**Supplementary data**

**Benralizumab for Adults with Rare and Off-Label Eosinophilic disorders: a 52-week Prospective, Single-Center Study**

Aviv Talmon, MD ^1, *^, Oded Shamriz, MD, PhD ^1,2^**^, *^,** Limor Rubin, MD ^1^, Yaarit Ribak, MD^1^, Iris Aynor ^1^,Adam Nevo, MD^3^, Anna Elia, MD^4^, Meitav Ben Sion, MD^3^, Esther Forkosh, MD^5^, Alon Hershko, MD, PhD^1^, Yuval Tal, MD, PhD ^1+^

| **Secondary Objective:** | **Outcome Measure:** |
| --- | --- |
| *To examine the clinical, laboratory and histological efficacy of benralizumab in non-asthma eosinophilic disorders following 48 weeks of benralizumab treatment* | *1.Improvement in one or more of the following clinical markers under benralizumab treatment as compared to the patient baseline status:*  *1.1* ***Non-disease specific clinical efficacy endpoints***   1. *Number of disease exacerbations during the study period, as compared to baseline.* 2. *Number of hospitalizations during the study period, as compared to baseline.* 3. *Decrease in dose of systemic GCs prescribed following benralizumab treatment initiation. At trial screening, baseline GCs dose and duration of treatment given to the patient will be defined by a senior clinical immunologist. Following benralizumab initiation, GCs will be tapered down. Clinical and laboratory markers (such as CRP and ESR) will be used to monitor patient reaction to GCs decrease including flare-ups.*   *1.2.* ***Disease-specific clinical efficacy endpoints***  ***a) Eosinophilic fasciitis:***   1. *Alleviation of weight loss (increase of 10% body weight in 48 weeks treatment period).* 2. *Resolution of findings in physical examination, as compared to images taken before treatment initiation.* 3. *Resolution of radiological image on MRI.*   ***b) Eosinophilic cellulitis:***   1. *Alleviation of severity and amount of skin lesions on physical examination following 48 weeks of benralizumab treatment, as compared to findings in physical examination at study initiation.*   ***c)Eosinophilic pneumonia and eosinophilic bronchiolitis:***   1. *Improvement in physical examination, spirometry studies (increase of 15% in forced expiratory volume in 1 sec) or resolution of severity and quantity of lesions seen in chest imaging (X-ray and computed tomography scans).* 2. *Improvement in systemic manifestations including reduction of severity and quantity of night sweats and increase in 10% of body weight following 48 weeks of benralizumab treatment.*   ***d)Eosinophilic cystitis:***   1. *Alleviation of urinary symptoms including hematuria and proteinuria.* 2. *Improved bladder emptying on ultrasound and cystography studies.* |

**Supplementary table 1: Secondary objectives and outcome goals of the study**

GCs- Glucocorticosteroids; CRP-C-reactive protein; ESR- Erythrocyte Sedimentation Rate; MRI- Magnetic resonance imaging

| **Liver enzymes** | | | | **Renal** | **CBC** | | | | | **Patient** |
| --- | --- | --- | --- | --- | --- | --- | --- | --- | --- | --- |
| **ALKP**  **30-120 U/L)** | **GGT**  **(0-55 U/L)** | **ALT**  **(0-45 U/L)** | **AST**  **(0-35 U/L)** | **Creatinine**  **(0.51-0.95 mg/dL)** | **ALC**  **(1.0-4.8 x 10^3^/L )** | **ANC**  **(1.8-7.7 x 10^3^/L )** | **WBC**  **(4.5-11.0 x 10^3^/L)** | **Platelets**  **(150-450 x 10^3^/L )** | **Hemoglobin**  **(13.5-17.5 gr/dL)** |  |
| 32 | 13 | 11 | 18 | 0.48 | 2.0 | 3.8 | 6.3 | 247 | 12.8 | 1 |
| NA | 41 | 48 | 47 | 0.93 | NA | NA | NA | NA | NA | 2 |
| NA | NA | NA | NA | NA | 2.7 | 2.3 | NA | 255 | 12.9 | 3 |
| NA | NA | NA | NA | 1.44^+^ | 0.99 | 4.73 | 6.4 | 221 | 11.4 | 4 |
| NA | 123 | 60 | 59 | NA | NA | NA | NA | NA | NA | 5* |
| 73 | NA | 18 | 14 | 0.73 | 2.47 | 2.92 | 5.9 | 268 | 12.6 | 6 |
| NA | 14 | 17 | 26 | 0.59 | 2.01 | 4.48 | 7.3 | 274 | 14.7 | 7 |
| 84 | 30 | 16 | 18 | 2.91^+^ | 2.31 | 7.01 | 10.1 | 329 | 15.1 | 8 |
| 58 | NA | 53 | 33 | 0.82 | 1.6 | 3.6 | 5.8 | 306 | 15.7 | 9 |
| 37 | NA | 30 | 26 | 0.5 | 1.8 | 2.4 | 4.7 | 178 | 13.2 | 10 |
| 92 | NA | 13 | NA | 0.6 | 1.5 | 3.1 | 5.1 | 334 | 11.5 | 11 |
| 68 | 12 | 15 | 17 | 0.86 | 4.2 | 6.8 | 11.9 | 255 | 16.5 | 12 |
| 56 | NA | 28 | 23 | NA | 1.9 | 4.1 | 6.6 | 223 | 16.6 | 13 |
| 65 | NA | 10 | NA | 0.7 | 2.27 | 3.72 | 6.4 | 322 | 11.7 | 14 |
| NA | 55 | 17 | 12 | NA | 4.58 | 5.87 | 11.5 | 447 | 13 | 15 |
| NA | NA | NA | NA | NA | 2.85 | 3.23 | 7 | 321 | 12.7 | 16 |
| 85 | NA | 25 | 29 | NA | 1.4 | 3.3 | 5.6 | 228 | 14.4 | 17 |

**Supplementary table 2: Complete blood count, liver enzymes and creatinine levels on last follow-up**

CBC- complete blood count; WBC- White blood count; ANC- Absolute neutrophil count; ALC- Absolute lymphocyte count; AST- Aspartate aminotransferase; ALT- Alanine transaminase; GGT- Gamma-Glutamyl Transferase; ALKP - Alkaline Phosphatase*P5 discontinued the treatment due to mildly elevated liver enzymes. ^+^ Baseline creatinine levels of P4 and P8 are 1.40 and 2.60 mg/dL, respectively.

AST-Aspartate aminotransferase; ALT- alanine transaminase; GGT- Gamma-glutamyl Transferase; ALKP- Alkaline Phosphatase; WBC- White blood cells; ANC- Absolute neutrophil count; ALC- absolute lymphocyte count; NA- data is not available.
